# Acute kidney injury: current concepts and new insights

**DOI:** 10.5249/jivr.v8i1.610

**Published:** 2016-01

**Authors:** Yavuzer Koza

**Affiliations:** ^a^Department of Cardiology, Ataturk University Faculty of Medicine, Erzurum, Turkey.

**Keywords:** Acute kidney injury, Epidemiology, Biomarkers, Management

## Abstract

**Background:**

Acute kidney injury, which was previously named as acute renal failure, is a complex clinical disorder and continues to be associated with poor outcomes. It is frequently seen in hospitalized patients, especially in critically ill patients. The primary causes of acute kidney injury are divided into three categories: prerenal, intrinsic renal and postrenal. The definition and staging of acute kidney injury are mainly based on the risk, injury, failure, loss, end-stage kidney disease (RIFLE) criteria and the acute kidney injury network (AKIN) criteria, which have previously been defined. However the clinical utility of these criteria is still uncertain. Several biomarkers such as Cystatin C and neutrophil gelatinase-associated lipocalin have been suggested for the diagnosis, severity classification and most importantly, the modification of outcome in acute kidney injury.

**Methods:**

Current literature on the definition, biomarkers, management and epidemiology of acute kidney injury was reviewed by searching keywords in Medline and PubMed databases.

**Results:**

The epidemiology, pathophysiology and diagnosis of acute kidney injury were discussed. The clinical implications of novel biomarkers and management of acute kidney injury were also discussed.

**Conclusions:**

The current definitions of acute kidney injury are based on the RIFLE, AKIN and KDIGO criteria. Although these criteria have been widely validated, some of limitations are still remain. Since acute kidney injury is common and harmful, all preventive measures should be taken to avoid its occurrence. Currently, there is no a definitive role for novel biomarkers.

## Introduction

Acute kidney injury (AKI) is a complex clinical disorder that is associated with severe morbidity and mortality. Despite technological advances in renal replacement therapy, AKI continues to be associated with poor outcomes. AKI is a syndrome of sudden loss of the kidney's excretory function, often with oliguria, which usually occurs over the course of hours to days. AKI is common in hospitalized patients, especially in critically ill patients.^1^ In the majority of patients, recovery of the kidney function is usually seen; however, many patients remain dialysis-dependent or are left with severe renal impairment.^1,2^ Since severe AKI is associated with a high mortality rate, all preventive measures should be taken to avoid the heavy burden of this common, but usually overlooked, clinical entity.

**Epidemiology**

In the Kidney Disease Improving Global Outcome (KDIGO) clinical practice guidelines, AKI is defined as any of the following: increase in serum creatinine (sCr) by≥0.3 mg/dl (≥26.5 umol/l) within 48 hours; or an increase in serum creatinine to ≥1.5 times baseline, which is known or presumed to have occurred within the preceding 7 days; or a urine volume <0.5 ml/kg/h for 6 hours. ^3^ The definition and staging of AKI are based on the risk, injury, failure, loss, end-stage kidney disease (RIFLE) criteria ^4^ and the acute kidney injury network (AKIN) criteria,^5^ which have previously been defined. 

The incidence of AKI varies according to the different patient populations, differences in parameters used for the criteria and timing of end points. In a population-based study of AKI using the RIFLE criteria, the annual incidence of AKI was 2147 per million population.^6^ This study examined hospitalized patients in whom only sCr had been measured. In another community study, the annual incidence of non-dialysis-requiring and dialysis-requiring AKI were respectively 3841 and 244 per million population.^7^ However, the criteria used for the diagnosis of AKI were different from the RIFLE criteria, and the baseline creatinine was derived from lowest creatinine during admission.

Recent hospital studies have reported that 3.2-21 % of all hospitalized patients and up to 50% of patients admitted to the intensive care unit develop AKI.^8-10^

**Pathophysiology**

AKI is now considered to be a broad clinical syndrome encompassing various etiologies, including acute tubular necrosis, pre-renal azotemia, acute interstitial nephritis, acute glomerular and vasculitic renal diseases, and acute postrenal obstructive nephropathy. Some of these conditions may coexist in the same patient.^4,5^ Impaired renal blood flow can lead to hypoxic injury to the renal tubular cells by depleting intracellular ATP, disrupting the intracelular calcium homeostasis, infiltration of leukocytes, injuring the endothelium, releasing cytokines and adhesion molecules and causing apoptosis.^11^ However, this ischemic cascade has little clinical relevance to illnesses such as sepsis.^12^ Another important characteristic of this ischemic model is the little relevance to periods of decreased perfusion, as can be seen during major surgery, since 80% renal-artery occlusion for 2 h does not lead to sustained renal dysfunction.^13^

In AKI, the renin-angiotensin-aldosterone system, the renal sympathetic system, and the tubuloglomerular feed back system are activated. These circulatory changes induce renal vasoconstriction and lead to increased release of arginine vasopressin, which contributes to water retention.^14^

**Diagnosis**

The clinical evaluation of AKI includes a careful history and through physical examination. Since there is no a specific symptom or a sign for AKI, it is usually diagnosed in the context of another acute illness. The most common sign is oliguria, but it is neither specific nor sensitive.^15^ sCr and urea concentrations are the most widely used parameters. In patients with increased sCr concentrations, it is important to distinguish whether the patient has AKI, chronic kidney disease, or a bout of acute illness superimposed on a chronic disease. In this context, some diagnostic clues that suggest the presence of chronic kidney disease may be helpful, namely, abnormal sCr prior to presentation, associated risk factors (eg, hypertension or diabetes), a slow clinical course for presenting illness and normocytic anaemia. Renal ultrasonography may provide evidence of chronic disease with small kidneys.^16^

In some cases, AKI occurs secondary to inflammatory parenchymal diseases such as vasculitis, glomerulonephritis and interstitial nephritis. In such patients, the clinical features of these diagnoses including systemic manifestations in vasculitis, the presence of macroscopic haematuria in glomerulonephritis and/or the recent initiation of treatment with a drug known to cause interstitial nephritis should be considered. Malignant hypertension, bilateral cortical necrosis, pyelonephritis, amyloidosis and nephrotoxins are well known other causes of parenchymal AKI.^16^

In the absence of obstruction or a clear prerenal cause, urinary microscopy with haematuria, proteinuria or fragmented red cells, red-cell casts, white cell casts or granular casts and any combination of these factors considerably suggests glomerular pathological changes. Although the sensitivity of the test is poor, urine samples should be tested for eosinophils in the suspicion of interstitial nephropathy.^16^

sCr concentrations and plasma urea concentrations are insensitive markers of glomerular filtration rate, as they are modified by nutrition, gastrointestinal bleeding, corticosteroid therapy, high protein diet, muscle mass, age, sex and aggressive fluid resuscitation.^16,17^ Indeed, increased levels of these waste products are observed only when the glomerular filtration rate decreases by more than 50% and does not show dynamic changes in filtration rates. Due to the limitations of sCr mentioned above, it is not an ideal marker for renal function. However, the sCr level is highly associated with the outcome in patients with AKI.^18^

**Drugs and AKI**

In drug-induced AKI, many patients present with a polyuric state, and thus, a high index of suspicion is needed for the diagnosis.^16,17^ Some of frequently prescribed drugs such as aminoglycosides, amphotericin, non-steroidal anti-inflammatory drugs, methotraxete, cisplatin, ciclosporin, angiotensin-converting-enzyme inhibitors and angiotensin-receptor blockers seem to be responsible for AKI in roughly 20% of patients, especially in critically ill patients.^19^

An important cause of AKI is the use of iodinated contrast agents in the diagnostic procedures such as angiography.^20^ Contrast-induced nephropathy can be prevented by use of iso-osmolar agents and isotonic saline infusion.^20,21^

**Novel Biomarkers**

As mentioned above, the creatinine level does not detect AKI promptly. Over the past decade, the discovery and validation of unique biomarkers of kidney injury has gained significant interest. Among these biomarkers, neutrophil gelatinase-associated lipocalin (NGAL) and Cystatin C are the most frequently studied. These promising markers seem to change earlier than sCr concentrations do, by showing different aspects of renal injury. For example, Cystatin C concentrations are related to changes in glomerular filtration rate,^22^ whereas concentrations of NGAL are related to tubular stress or injury.^23^ Changes in these biomarkers with treatment or recovery suggest that they can also be used to monitor interventions.^24^ Furthermore, they can distinguish a majority of patients who do not have AKI according to creatinine-based criteria, but actually have a degree of kidney stres or injury that is associated with worse outcomes.^25^

Cystatin C is now considered a superior marker when compared with sCr in both animal models and clinical settings of chronic kidney disease.^26,27^ However, it is unclear whether the value of cystatin C is generarizable to all forms of AKI or not. Moreover, the analysis of cystatin C is affected by diabetes, hyperthyroidism, inflammation, large doses of corticosteroids, hyperbilluribinemia, rheumatoid factor and hypertrigliseridemia.^28^

NGAL is the most extensively studied renal biomarker and it has been demonstrated in a recent meta-analysis that serum and urine NGAL levels have been found to be not only diagnostic of AKI, but that they have also predicted the clinical outcomes, such as the need for inititaion of dialysis, and mortality.^25^

To date, several other biomarkers such as microalbumin, N-acetyl-B-D-glucosaminidase, kidney injury molecule-1, interleukin-18, liver fatty acid-binding protein, netrins and nestin have been studied for the diagnosis, severity classification and most importantly, the modification of the outcome in AKI.^29,30^ However, more clinical studies will be required to prove the true superiority and cost effectivity of novel biomarkers over creatinine.

**General Management**

Since there is no an established pharmacotherapy for AKI, all preventive measures should be taken to prevent its occurrence. For example, if pre-renal factors contribute, they should be identified and rapid administration of intravenous fluids should be quickly undertaken. In this regard, the association between a positive fluid balance and increased 60-day mortality should be kept in mind.^31^ In fluid-resuscitated critically ill patients with pronounced oliguria or anuria, the avoidance of fluid overload can be provided by the initiation of renal replacement therapy at an early stage.^16^

Central volume status can be monitored by physical examination, central venous pressure and measurement of blood pressure and heart rate.

Nutritional support should be started as early with adequate calories, protein, trace elements and vitamins.^17^

Hyperkalemia should be treated with insulin, dextrose, a bicarbonat infusion and/or nebulised salbutamol. If the serum potassium concentration is higher than 7 mmol/L or if electrocardiographic signs of hyperkalemia are present, 10 ml of 10% calcium gluconate should also be given intravenously.^16,17^

As the nephroprotective effect of renal-dose or low dose dopamine has been refuted by findings from several systematic reviews, use of this strategy is not recommended.^17,32,33^

Although loop diuretics such as furosemide and bumetanide are commonly used in the management of AKI, their use are not recommended for the prevention or treatment of AKI, except in the management of volume overload.^17^

**Renal replacement therapy**

When making the decision for renal replacement therapy (RRT), the clinicians must consider some factors such as potassium, creatinine, and urea concentrations; fluid status; urine output; the overall course of the patient's illness; and the presence of other complications.

Absolute indications for initiation of RRT:^16^

1. Anuria (negligible urine output for 6h)

2. Severe oliguria (urine output<200 ml over 12h)

3. Hyperkalemia (potassium concentration >6.5 mmol/L)

4. Severe metabolic acidosis (pH<7.2 despite normal or low partial pressure of carbon dioxide in arterial blood)

5. Volume overload (especially pulmonary odema unresponsive to diuretics)

6. Pronounced azotemia (urea concentrations >30 mmol/L or creatinine concentrations >300 umol/L)

7. Clinical complications of uremia (eg, encephalopathy, pericarditis, neuropathy)

Due to the fact that the only studies linking timing with the outcome are observational, the optimal time to start RRT is still debatable.^34,35^ The available forms of RRT include: continuous, intermittent and peritoneal dialyses. Continuous RRT includes filtration alone (ie, continuous venous-venous haemofiltration) or diffusion alone (eg, continuous veno-venous haemodialysis), or both (eg, continuous veno-venous haemodiafiltration).^16^

Since there are no randomised controlled trials comparing intermittent or continuous RRT intermittent haemodialysis, slow low efficiency dialysis, with continuous RRT, all seem to be acceptable options.^36^

As it is unclear, the Acute Renal Trial Network (ATN)^37^ and Randomised Evaluation of Normal versus Augmented Level of Renal Replacement Trial (RENAL)^38^ studies have been designed to investigate the appropriate intensity of RRT. Both have shown no difference in survival rates with increasing intensity of RRT. The current evidence suggests that the prescribed dose of RRT should be equivalent to 20-30 ml/kg/h of continuous RRT or intermittent RRT three times weekly. This requires careful monitoring as there is often a significant reduction in the dose delivered versus prescribed. Hemodynamically unstable patients should preferably receive continuous RRT.^37,38,39^

## Conclusion

Since AKI is common, harmful and treatable, all efforts should be focused on minimising the causes of AKI, on increasing awareness of the importance of serial measurements of sCr in high risk patients, and on documenting the urine volume in acutely ill patients in order to achieve early diagnosis; there is as yet no definitive role for alternative biomarkers.
